# Metacommunity Dynamics: Decline of Functional Relationship along a Habitat Fragmentation Gradient

**DOI:** 10.1371/journal.pone.0011294

**Published:** 2010-06-30

**Authors:** Benjamin Bergerot, Romain Julliard, Michel Baguette

**Affiliations:** 1 MNHN-CNRS-UPMC, UMR 7204 CERSP, Paris, France; 2 MNHN-CNRS, UMR 7179 MAOAC, Brunoy, France; 3 CNRS, USR 2936, Station d'Ecologie Expérimentale du CNRS, Moulis, France; University of Lancaster, United Kingdom

## Abstract

**Background:**

The metacommunity framework is crucial to the study of functional relations along environmental gradients. Changes in resource grain associated with increasing habitat fragmentation should generate uncoupled responses of interacting species with contrasted dispersal abilities.

**Methodology/Principal Findings:**

Here we tested whether the intensity of parasitism was modified by increasing habitat fragmentation in the well know predator-prey system linking the parasitoid *Cotesia glomerata* (Hymenoptera: Braconidae) to its main host *Pieris brassicae* (Lepidoptera: Pieridae). We collected information on herbivorous abundance and parasitism rate along an urbanization gradient from the periphery to the centre of Paris. We showed that butterfly densities were not influenced by habitat fragmentation, whereas parasitism rate sharply decreased along this gradient.

**Conclusions/Significance:**

Our results provide novel insights into the mechanisms underlying the persistence of species in highly fragmented areas. They suggest that differential dispersal abilities could alter functional relationships between prey and predator, notably by a lack of natural predators.

## Introduction

Populations of a species interact through two kinds of networks, firstly in food webs of co-occurring species within local communities (e.g. [1]), secondly as spatially structured local populations linked by dispersal of conspecific individuals within metapopulations (e.g. [2]). The metacommunity framework has cross-fertilized these two networks, a metacommunity being defined as a set of local communities that are linked by the dispersal of multiple interacting species [3]. Indeed, at a local scale, food webs are necessarily assembled by colonization and depleted by extinction [4]. Both colonization and extinction are influenced by spatial processes, as well as by the web of interactions defined by the local food web. In such a context, the federative nature of the metacommunity framework has the potential to illuminate research questions in the field of either communities and food webs or metapopulations from an innovative viewpoint. Here we use the metacommunity framework to get new insights in a crucial conservation topic, the persistence of functional relationships in prey/parasitoid systems across fragmented landscapes [5].

Fragmentation of natural habitats by human activities is usually considered as one of the major threats to biodiversity, by increasing the extinction rates of local populations (e.g. [6]–[8]). Landscape spatial structure, i.e. the spatial relationships among habitat patches and the matrix in which they are embedded, is of central importance in understanding the effects of fragmentation on population dynamics (e.g. [9]). Habitat fragmentation directly impacts landscape spatial structure by decreasing the total area of suitable habitats, which in turn alters habitat connectivity by increasing the distance between always smaller and more isolated patches [6], [10].

However, many studies report confounding patterns in the response to habitat fragmentation that corresponds to deviations from the expected positive species-area relationship predicting higher extinction probabilities with decreasing fragment area to various responses which deviate from this expected relationship [11]. This particular pattern has been explained by either the irruption of matrix-dwelling species in small fragments and/or the supplementation of fragment-dwelling species by matrix located resources [11]. An alternative explanation would be the alteration of functional relationships between interacting species due to their differential sensitivity to the fragmentation process. Indeed, some species are systematically disadvantaged in small or isolated habitats, so both the community structure and the species interactions with their environment will change [12].

Parasitoids are organized in clear communities where the potential number of interactions is temporally and spatially limited [13]. Such simple food webs are thus excellent models to understand how trophic relationships drive metacommunity structure and dynamics. In this study, we expect that two interacting species with contrasted dispersal abilities will show uncoupled spatial dynamics along a habitat fragmentation gradient, which in turn will affect their functional relationships [14].

To test this hypothesis, we designed an experiment using a simple tri-trophic system involving a plant (potted cabbages) –an herbivore (the butterfly *Pieris brassicae*)–and a predator (the parasitoid wasp *Cotesia glomerata*). We selected a couple herbivorous/parasitoid pair with contrasted dispersal abilities: maximal dispersal distance of several kilometers for the butterfly [15] *vs.* several hundred of meters for the wasp [16]. Traps containing caterpillars and their host plant were disposed at even intervals from the periphery to the center of the city of Paris, i.e. along a growing gradient of fragmentation of butterfly habitats (based on the presence of larval food plants). Parasitoids were free to penetrate into the traps, whereas caterpillars were unable to leave them. We investigated how the parasitism rates inside the traps were related to their position along the fragmentation gradient. As urbanization increases landscape fragmentation [10], here we used the urbanization gradient as a proxy of fragmentation. We also investigated the density of free flying adult *P. brassicae* along the same gradient.

## Materials and Methods

### Ethics Statement

All animal work had been conducted according to relevant national and international guidelines. Butterflies were reared at the National Museum of Natural History in Brunoy (France) under controlled conditions. We developed a harmless and specifically experimental protocol to trap parasitoids. All individuals were released after experiment. Observational and field studies were made in private properties.

### Parasitism rates

**Figure 1:**
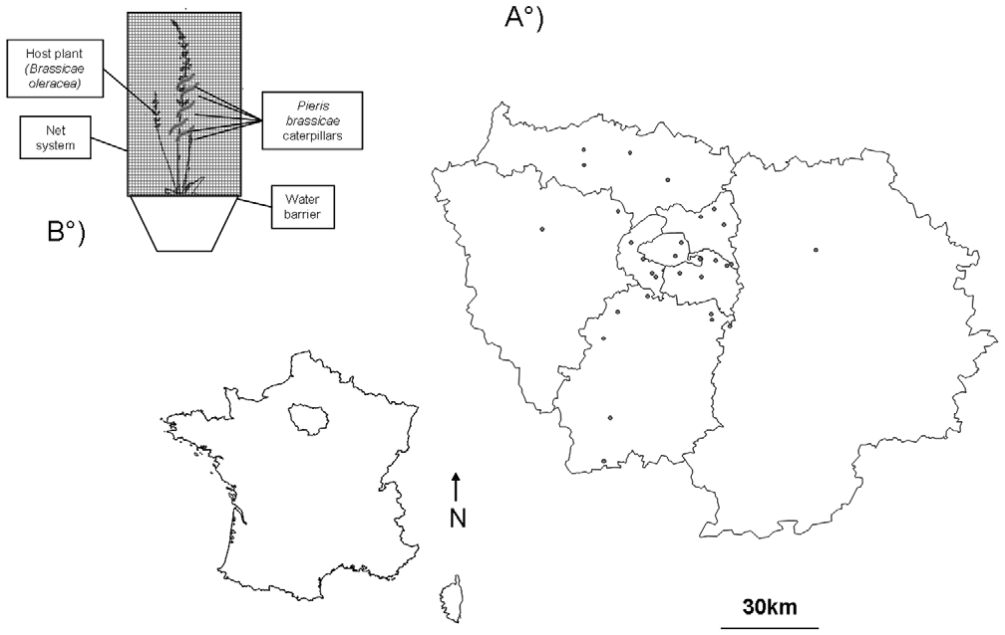
Map and location of the experimental trap. 1.A. Map of the Île-de-France region and locations of the study plots and B. Experimental trap used in the study.

*Cotesia glomerata* is a gregarious larval endoparasitoid of *Pieris brassicae* [17], [18]. Parasitoid data were obtained by trapping *Cotesia glomerata* using an original experimental protocol. Traps were placed at 30 sites arranged along a fragmentation gradient in the Île-de-France region that encompasses the city of Paris (Fig. 1A). They were placed in gardens with a surface at least equal to 10 m^2^ and where at least 20 crucifers were initially present (among them, we found species such as *Alliaria petiolata, Brassica oleraceae, Brassica rapa, Sinapis arvensis, Sinapis alba, Brassica nigra, Erysimumcheiri, Cardamine* spp., *Arabis hirsute*). These host plants were very common and widely represented in the Île-de-France region [19]. Indeed, the presence of potential host plants is crucial in parasitoid habitat selection as wasps use host plant odours to locate caterpillars [20]. The patch must be wide enough and contain butterfly host plants in order to attract parasitoids. Indeed, scents are emitted during plant attacks by caterpillars and these particular scents play an important part in host location by adult parasitoids wasps [21]. The access to private gardens was possible with the help of the French Butterfly Garden Observatory volunteers. They allowed us to choose 30 sites among hundred and selected the most appropriated.

Each trap contained a potted host plant (*Brassica oleracea* var. *gemmifera*, Zenker), 5–7 first instar *Pieris brassicae* caterpillars, a water barrier preventing caterpillar's escape. The trap was surrounded by a net allowing parasitism but not predation for example by birds (Fig. 1B). At least two traps were placed in each site each year but traps were placed only in 5 sites in 2008 due to coordination issues and in 30 sites in 2009. Thus, data from two years was lumped in the analyses. Traps were placed in sites at the beginning of august 2008 (from 8^th^ August to the end of experiment) and 2009 (from 1rst August to the end of experiment). This period was chosen because it matches the summer *Cotesia glomerata* emergence peak (Bergerot B., unpublished results).

*Pieris brassicae* caterpillars were reared in a laboratory and were arranged at their first stage in the trap. Larvae of *Pieris brassicae* were obtained in the laboratory by placing adult butterflies coming from the Île-de-France region in an oviposition cage (80×80×80 cm) with cabbage leaves (*Brassica oleracea* L.) under incandescent light to maintain a 14L: 10D photoregime. A sugar water solution (1:10 flower honey, 9: 10 water) in Eppendorf tube was provided as a carbohydrate and water source. To produce synchronized batches of young larvae, oviposition plants were changed every 2 days. Eggs on plants were held in a growth chamber at 23°C and 50% r.h. until larval hatch and fed for 1 day. Larvae were then used in the experiment.

Parasitism data were collected by daily observations from the installation of the trap to the end of the experiment (approximately 20 days). The end of the experiment was defined: (1) if parasitoid cocoons appeared, (2) if caterpillars died and (3) if caterpillars pupated. The parasitism rate was calculated as the number of parasitized caterpillars divided by the number of caterpillars that survived until cocoons may appeared, and the parasitoid virulence on pre-imaginal stage was the number of emerging parasitoid cocoons of *Cotesia glomerata*, both counted at the end of the experiment.

### Density of free flying butterflies

The instantaneous density of adult *Pieris brassicae* was estimated along the fragmentation gradient by counting the number of butterflies in each study plot during 10 minutes, once a week, simultaneously, during periods with a wind speed less than 5 on the Beaufort scale, air temperature ≥17°C and sunshine ≥75%. Only 25 (over 30) sites were taken into account to estimate butterfly density (number of individuals/m^2^) due to the possibility of butterfly overestimation in small gardens. *Pieris brassicae* is known to reproduce throughout the study area althougth it was not possible to assess reproduction in all study plot.

### Landscape fragmentation

Data were collected throughout the highly urbanized Île-de-France region (Fig. 1A), which shows strong structuring contrast at the landscape level. We extracted three main classes of ground covers from a GIS of the Soil Occupation Model classification database [22]: artificial urban cover (including 54 habitat classes such as buildings, parking or roads), open urban cover (including 14 habitat classes such as gardens) and rural cover (including 15 habitat classes like forest and crop fields). Only the two last categories provide suitable resources for the reproduction of the butterfly. To estimate the level of fragmentation, we defined a 1 km radius around each site, in which we calculated the proportion of each of three ground cover classes. This 1 km range was chosen because *Pieris* butterflies can escape from their predator, *Cotesia glomerata*, by colonising new habitats at distances of at least 1 km [23]. We then looked for possible correlations between the ground cover categories. We found a significant (negative) correlation between artificial urban and rural areas R = −0.67, df = 28, p<0.001). Accordingly, we used only open urban areas and artificial urban areas in subsequent analyses (both variables were statisticaly not correlated). These two variables were considered as representative for the habitat fragmentation gradient. We also calculated the proportion of urban areas in concentric rings (0–250 m, 250–500 m, 500–1000 m, 1000–2000 m, 2000–3500 m and 3500–5000 m) in order to examine the impact of the level of urbanization at more than one spatial scale. We then looked urbanization thresholds by looking for correlations between each urbanization level at each radius.

### Statistical analyses

Generalized Linear Models (GLMs) were used to test if parasitism was related to ground cover and to adult butterfly density. We modeled the proportion of parasitized caterpillars using GLM (pooling traps for a site) with the surface of open urban areas, the surface of artificial urban areas, the density of free flying butterflies and their interactions as explanatory variables and assuming a binomial error. We used a stepwise selection procedure to select the best fitted model based on the Akaike Information Criterion, AIC [24]. The selection process was based on both backward/forward stepwise regression search, which involves starting with all candidate variables and testing for statistical significance one by one. Analyses of variance (ANOVA) of the GLMs were made using a type 3 ANOVA and associated P-values were calculated. The contribution for each independent variable in the best model fitted was calculated by applying the hierarchical partitioning algorithm [25]. Parasitoid virulence was analysed by a one-way analysis of variance (ANOVA) with site as a covariate. Habitat effects on butterfly densities were investigated using GLMs assuming a Poisson distribution for the counts an using the two ground cover variables and their interactions as explanatory variables. All statistical analyses were performed with R2.7.0©.

## Results

**Figure 2:**
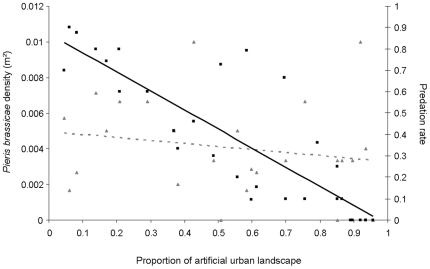
Uncoupled responses of butterfly density and parasitism rate to urbanization. Relationship between the proportion of artificial urban landscape and A. *Pieris brassicae* density (number of individuals/m^2^) and B. the parasitism rate. Black squares figure the relation between parasitism rate and proportion of artificial urban landscape and full black and bold line represents linear regression these two variables (R = −0.85). Pale grey triangles figure the relation between the proportion of artificial urban landscape and *Pieris brassicae* density and dotted grey line represents linear regression these two variables (R = −0.18).

The best model explaining the parasitism rate included artificial urban areas and *Pieris brassicae* density without any interaction (Table 1). The parasitsim rate was significantly negatively related both to the artificial urban area (*LR Chisq* = 123.34, p<0.001, Fig. 2) and to *P. brassicae* density (*LR Chisq* = 6.88, p = 0.01). Artificial urban area explained 96.19% of the total variance in the model while *Pieris brassicae* density explained the remaining 3.81%. The parasitoid virulence (mean number of parasitoid cocoons per caterpillar = 21.36±2.20) did neither significantly differ among sites (F_23, 149_ = 0.71, p = 0.83) nor according to artificial urban area (F_1, 22_ = 0.63, p = 0.44). The stepwise selection procedure showed that none of the two ground cover variables was significantly related to *P. brassicae* densities (df = 1, open urban areas, *LR Chisq* = 0.20, p = 0.65; artificial urban areas, *LR Chisq* = 0.36, p = 0.55, Fig. 2).

**Table 1:** Models tested using the GLM procedure and the associated Akaike Information Criterion (AIC) obtained by backward stepwise selection procedure.

Model tested in GLM procedure	AIC
Parasitism ∼ Artificial urban cover * Open urban cover + Pieris density * Artificial urban cover + Pieris density * Open urban cover	115,8
Parasitism ∼ Artificial urban cover * Open urban cover + Pieris density * Open urban cover	113,8
Parasitism ∼ Artificial urban cover * Open urban cover + Pieris density	111,9
Parasitism ∼ Artificial urban cover + Open urban cover + Pieris density	110,09
Parasitism ∼ Artificial urban cover + Pieris density	108,4

All proportions of artificial urban areas calculated at different spatial scales were strongly correlated (Table 2). Accordingly, we did not detect any threshold in the urbanization gradient.

**Table 2:** Correlations between the percentages of artificial urban areas calculated in concentric rings at various landscape scales (in meters).

	0–250 m	250–500 m	500–1000 m	1000–2000 m	2000–3500 m	35000–5000 m
0–250 m	1	0.57**	0.63***	0.47**	0.42*	0.43*
250–500 m		1	0.9***	0.81***	0.7***	0.72***
500–1000 m			1	0.85***	0.8***	0.79***
1000–2000 m				1	0.9***	0.9***
2000–3500 m					1	0.97***
3500–5000 m						1

The asterisks show significant Pearson correlations coefficients (*P<0.05, **P<0.01, ***P<0.001).

## Discussion

We analyzed the parasitism rate of *Cotesia glomerata* on *Pieris brassicae* caterpillars and the density of adult butterflies along a gradient of increasing habitat fragmentation from the periphery to the centre of Paris. The parasitism rate linearly decreased with increased artificial urban areas around the study plots, the proportion of parasitized caterpillars falling from 90% to 0% when the proportion of urban areas increased from 10% to 90%. By contrast, the parasitoid virulence (the number of parasitoid cocoons per parasited caterpillar) remained constant all along the fragmentation gradient, which suggests no alteration in the parasitoid efficiency. The density of adult butterflies was not affected by the fragmentation gradient, although the parasitism rate decreased with increasing butterfly densities.

A real measure of parasitoid virulence would be the number of cocoons per caterpillar per parasitoid. However, *Cotesia glomerata* deposit 15–35 eggs by caterpillar [26]. With a mean number of around 21 parasitoid cocoons per caterpillar, our results suggested that only one female layed per caterpillar. A higher density per host caterpillar would have various costs such as small body size and high mortality [27], suggesting that *Cotesia* glomerata females avoid laying in previously parasited host. Accordingly, we are confident that female virulence was unaffected by the fragmentation gradient.

As mentioned in the literature (e.g. [28]) and not surprisingly, *Cotesia glomerata*, which occurs at the third trophic-level of the community, was more sensible to habitat fragmentation than *Pieris brassicae*. Indeed, abundance and diversity of parasitoids were often more strongly affected by habitat fragmentation than the abundance and diversity of herbivorous hosts, even at the scale of few hundred meters [29]. More generally, parasitoids were more sensitive to urbanization than their hosts [12], [30], [31] and the absence of higher trophic levels can affect the population dynamics of lower levels and even the stability of the trophic system as a whole [32].

Recent plant-insect community studies showed that interactions between species were influenced either by factors in the local patch or by factors from the surroundings [33]. Parasitoids were notably affected by the average isolation of their habitats and the diversity of these habitats in the surroundings [34]. In our study, as the proportions of urban areas were strongly correlated at different spatial scales, the surrounding context of population dynamics did not differ. However, such analysis is crucial because it allows identifying the spatial scale that has the largest influence on population dynamics [35]. Indeed, for some interacting species, the surrounding contexts of population dynamics were found to differ because the dynamics of some species depend on processes acting on small scales of their surroundings, whereas others species react processes acting at larger parts. Accordingly, landscape fragmentation does not affect all species in a similar way with notable consequences on food web interactions [12].

Concerning butterfly sensitivity to fragmentation, previous studies had similarly reported that the incidence and the density of some butterfly species (including *Pieris brassicae*) were not affected by a habitat fragmentation in urbanized areas [36]. Three main hypotheses could explain this pattern. Firstly, the putative increase of butterfly larval food plants in highly urbanized areas is worth considering [12], [36], but not supported by specific field data in this study. In our case, in suburban landscapes, *Pieris brassicae* was regularly observed on cabbage species (such as *Brassica oleracea L*.) and many cultivar and cruciferous weeds were well represented each study site includes at least 20 cruciferous plants (spontaneous in 90% of the cases). Secondly, source-sink functioning should also be considered. Populations in urbanized and fragmented landscapes could be permanently reinforced by individuals coming from the periphery. However, adults of *P. brassicae* reared from eggs laid in urbanized areas have significantly higher mobility performances than those coming from rural landscapes and these differences seem to have a genetic basis (S. Ducatez, unpubl. results).

Interacting species may also differ substantially in their dispersal rates and ranges. Thus, they all experience a different spatial structure of the habitat in the same landscape [37]. As the distribution of a given plant species can be viewed as a single large patchily distributed population if dispersal is frequent [14]. This plant may feed a herbivore that forms a classic metapopulation made up of local populations with relatively independent dynamics; and this herbivore may in turn have several functionally important predators and parasites, each with their own relationship to the spatial structure of the host populations with which they interact. While the spatial distribution of habitats will play some role in any species interaction, it is especially significant in those situations where both the relative dispersal rates and distances of interacting species differ greatly.

Our results show that the third-trophic level species represented by *Cotesia glomerata* has indeed a smaller distribution range than the lower trophic level species [29], represented by *Pieris brassicae* and their host plants. Several studies have suggested also that fragmentation could differentially affect insects in different guilds and trophic levels, potentially disrupting metacommunity functioning [38], [39]. In general, higher levels of habitat fragmentation lead to increased herbivore incidence, partly because parasitoids can only colonize patches already occupied by their hosts [28] and also probably because they do not disperse as well as their host. Modest degrees of isolation of suitable patches within a metapopulation can contribute to the stability of the system because this offers to herbivores the opportunity to escape temporally from parasitoids by continuously colonizing new habitat patches. However, large scale fragmentation and the resulting isolation of the patches, can lead to the destabilization of multi-trophic systems, and hence of metacommunity functioning because parasitoids will be absent in many isolated patches [32]. Yet, few studies have mentioned so far that the relaxation of the parasitism rate with increasing habitat fragmentation that we document here.

This process could contribute to the persistence of prey species in highly fragmented landscapes. The most parsimonious explanation of this difference is the contrasted dispersal abilities of the parasitoid and the prey (several kilometers for the butterfly [15] *vs.* several hundred of meters for the parasitoïd [16]). Given this huge difference, we expect that the grain size of the landscape, i.e. the spatial scale at which the parasitoid and the prey will be able to react to spatial heterogeneity [40] will differ from at least two orders of magnitude. In other words, functional distances between local populations of a *Cotesia* metapopulation will be at least hundred times lower than those of a *Pieris* metapopulation, which will induce a completely uncoupled response of the parasitoid and the prey to the fragmentation gradient. Given that in agricultural landscapes, populations are frequently able to persist under parasitism rates of 60–80% [15], [17], we investigated how the parasitism rate was relaxed according to habitat fragmentation. When habitat patches are surrounded by at least 30% of urban structures within a 1 km radius (Fig. 2), the parasitism rate fell under 60%.

In this study, lower trophic levels represented by host plants and butterflies were present in all sites. Thus, we could easily suggest parasitoids were less present in urban areas due to their low dispersal abilities. However, we cannot rule out other synergistic effects that could have reinforced the uncoupled spatial dynamics of the parasitoid and its host caused by unequal dispersal abilities. Herbivory induces the emission of plant volatiles that have an important part in host location by adult parasitoid wasps (e.g. [21]). Yet, volatile emissions by plants vary with fluctuating abiotic parameters such as barometric pressure, humidity and light conditions [41]. Thus we could suppose that landscape structure and pollution (air pollution [42], or light for example [43] might affect plant volatile detection by parasitoids seeking for their hosts.

There is one methodological caveat to our study design. Samples were mainly restricted only to one year (2009). Only five sites were sampled in 2008 and were lumped with 2009 data. Thus, the question of reproducibility across years remains open. In 2008 and 2009, local and landscape structures was not modified in our study sites. Thus, at a smaller scale, we expected (and checked) that parasitism rate did not show significant difference between the five sites in 2008 and 2009 (Chi-square test, χ^2^ = 3.05, df = 4, p = 0.55). However, if no trend modification was apparent in this study, we could expect differences and hence significant variation of interactions between species across year if perturbation occurred across year at local scale (site) and more globally at the landscape scale [44].

To conclude, we expect that such alteration of functional relationships by differences in dispersal ability between species will have strong consequences on the functioning of metacommunities. Many metacommunity studies focus on the spatial variation of incidence and density of species belonging to the same taxonomic group but neglect trophic interactions. Our results suggest that these trophic interactions might be among the key factors in such spatial pattern variability.
